# Tyrosol Prevents Ischemia/Reperfusion-Induced Cardiac Injury in H9c2 Cells: Involvement of ROS, Hsp70, JNK and ERK, and Apoptosis

**DOI:** 10.3390/molecules20033758

**Published:** 2015-02-25

**Authors:** Liwei Sun, Hang Fan, Lingguang Yang, Lingling Shi, Yujun Liu

**Affiliations:** National Engineering Laboratory for Tree Breeding, College of Biological Sciences and Biotechnology, Beijing Forestry University, 35 Qinghua East Road, Haidian District, Beijing 100083, China; E-Mails: Lsun2013@bjfu.edu.cn (L.S.); fwqh1990@163.com (H.F.); Yanglingguangxdjqz@163.com (L.Y.); linglingshi2005@163.com (L.S.)

**Keywords:** tyrosol, ROS, Hsp70, JNK, ERK, Bcl-2/Bax ratio, caspase-8, apoptosis

## Abstract

Ischemia-Reperfusion (I/R) injury causes ROS overproduction, creating oxidative stress, and can trigger myocyte death, resulting in heart failure. Tyrosol is an antioxidant abounded in diets and medicine. Our objective was to investigate the protective effect of tyrosol on I/R-caused mortality in H9c2 cardiomyocytes through its influence on ROS, Hsp70, ERK, JNK, Bcl-2, Bax and caspase-8. A simulated I/R model was used, myocytes loss was examined by MTT, and ROS levels were measured using DCFH-DA. Nuclear condensation and caspase-3 activity were assessed by DAPI staining and fluorometric assay. Phosphorylated ERK and JNK were determined by electrochemiluminescent ELISA, and Hsp70, Bcl-2, Bax and caspase-8 were examined by Western blotting. Results show that tyrosol salvaged myocyte loss, inhibited nuclear condensation and caspase-3 activity dose-dependently, indicating its protection against I/R-caused myocyte loss. Furthermore, tyrosol significantly inhibited ROS accumulation and activation of ERK and JNK, augmenting Hsp70 expression. Besides, tyrosol inhibited I/R-induced apoptosis, associated with retained anti-apoptotic Bcl-2 protein, and attenuated pro-apoptotic Bax protein, resulting in a preservation of Bcl-2/Bax ratio. Finally, tyrosol notably decreased cleaved caspase-8 levels. In conclusion, cytoprotection of tyrosol in I/R-caused myocyte mortality was involved with the mitigation of ROS, prohibition of the activation of ERK, JNK and caspase-8, and elevation of Hsp70 and Bcl-2/Bax ratio.

## 1. Introduction

Ischemic heart disease is the most common health problem around the world, leading to the cardiac infarction and mortality. Early reperfusion by surgical coronary intervention and thrombolysis is currently the most practical approach for relieving ischemia and can rescue myocytes from ischemia-caused cell mortality. However, reperfusion may self-contradictorily aggravate myocardial injury. This phenomena is termed as ischemia-reperfusion injury (I/R), which is to a large extent attributed to the disruption of normal oxidative metabolism. Oxidative metabolism is presented in the normal cell, and accumulation of reactive oxygen species (ROS) occurs at very low concentrations, which is attributable to the equilibrium of disposal rates by antioxidants and enzyme systems within, thus forming a stable balance [1]. However, this balance can be disrupted by many insults including I/R injury, leading to overproduction of ROS and creating oxidative stress [2,3]. This damages DNA, proteins, and membrane lipids and compromises cellular function and integrity [4,5], triggering cell mortality, apoptosis, and finally leading to cardiomyocyte loss and heart failure [6,7,8].

Hsp70 is an ubiquitous inducible stress protein highly expressed in cells under a large variety of stress insults to confer cytoprotection [9,10]. It works as a molecular chaperone and plays a pivotal role in maintaining and repairing cellular homeostasis or disruption under thermal, I/R and oxidative stress [11,12]. Numerous evidences have proved that overexpression of Hsp70 builds up cardiac resistance to I/R injury, thus delivering protection against myocardial impairment during I/R [13,14,15]. Furthermore, JNK transduction pathway and caspases cascade including caspase-8 are correlatively activated by oxidative stress, thus mediating the initiation of apoptosis and cell mortality [16,17,18], and the ERK transduction pathway plays a double-edged role in the determination of cellular survival or death in the event of apoptosis or necrosis [19,20,21,22,23,24,25]; However, its role in the H9c2 cardiomyocyte during I/R remains unclear. In addition, eventual vulnerability of cells to apoptotic stimulus including oxidative stress is determined by the decreasing relative ratio of anti-apoptotic and pro-apoptotic members of the Bcl-2 family (Bcl-2/Bax ratio), thus activating caspase-3 enzyme to initiate apoptosis [16,26].

Intervention targeted at inhibition of the loss of myocytes caused by oxidative stress during I/R injury is a potent gateway to counteract I/R-caused cardiac impairment. To date, bioactive compounds found in herbal medicine or diets have gained increased attention because of their relative nature, safety and prevalence. Tyrosol is a widespread bioactive phenol compound found abundantly in diets (olive oil [27,28], white wine [29] *etc.*) and cardioprotective herbal medicine such as rhodiola [30,31,32,33,34,35]. It has been reported that the majority of tyrosol was distributed in the heart after ingestion, implying its protective effect on the cardiomyocytes [36]. Reports have shown that tyrosol can improve intracellular antioxidant defense systems in macrophages and exert antioxidant capacity in human endothelial cells [37] and Caco-2 cells [38]. However, whether tyrosol can regulate oxidative stress-caused myocyte loss during I/R and the mechanisms remain to be fully elucidated. Therefore, the aim of present study is to investigate the protective effect of tyrosol on apoptosis and myocyte loss during I/R injury in H9c2 cardiomyocytes through its impact on ROS accumulation, Hsp70 expression, ERK and JNK transduction pathway, Bcl-2/Bax ratio, and the activation of caspase-8. 

## 2. Results and Discussion

### 2.1. Tyrosol Inhibited I/R-Caused Cell Death in Cardiomyocytes

**Figure 1:**
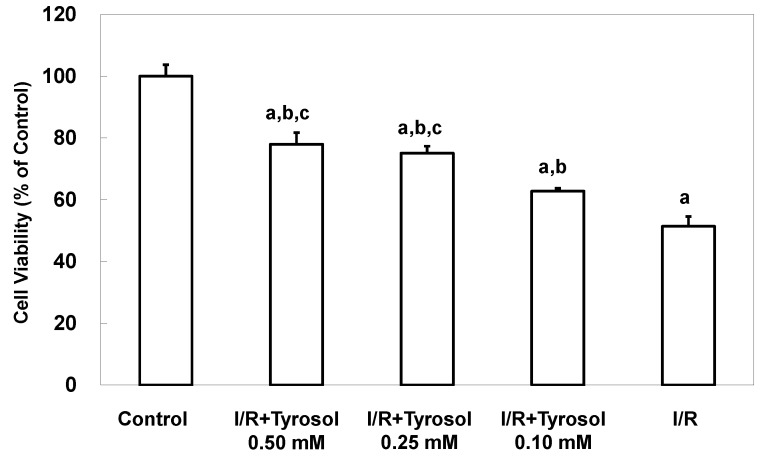
Effect of tyrosol on the viability of H9c2 cardiomyocytes. Cells were incubated overnight prior to treatment with concentrations of tyrosol for 24 h, then I/R model was performed in the presence of the same treatment with tyrosol, and the MTT assay was then performed. Results are expressed as percentages of the control value and the means ± SEs from 10 independent experiments are shown. a: *p* < 0.05 compared with control, b: *p* < 0.05 compared with I/R cells, c: *p* < 0.05 compared with I/R + Tyrosol 0.10 mM cells.

To examine protective effects of tyrosol on death of H9c2 cardiomyocytes during I/R, experiments were conducted using MTT assay. Figure 1 shows that I/R caused significant cell death, while tyrosol treatment of H9c2 cardiomyocytes mitigated I/R-induced cell death. Significance was exhibited between three treatment groups (I/R + Tyrosol 0.5 mM *vs.* I/R + Tyrosol 0.1 mM; I/R + Tyrosol 0.25 mM *vs.* I/R + Tyrosol 0.1 mM) (Figure 1).

**Figure 2:**
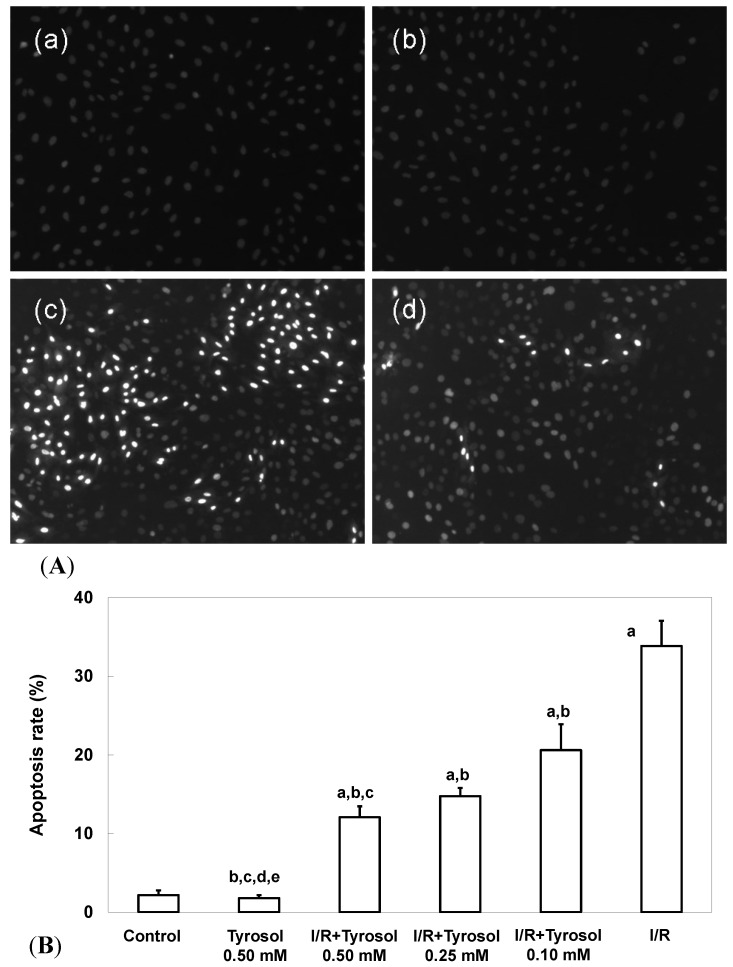
Effect of tyrosol on nuclear condensation in H9c2 cardiomyocytes following I/R. (**A**) Apoptosis associated with nuclear condensation in H9c2 cardiomyocytes. (a) control, (b) tyrosol 0.50 mM, (c) I/R, (d) I/R + tyrosol 0.50 mM. (**B**) Proportions of apoptosis among H9c2 cardiomyocytes. Results are expressed as percentages and the means ± SEs of the data of five independent experiments. Condensed nuclei in 20 randomly chosen microscopic fields from each sample were counted using the Image J software. a: *p* < 0.05 compared with control, b: *p* < 0.05 compared with I/R cells, c: *p* < 0.05 compared with I/R + Tyrosol 0.10 mM cells, d: *p* < 0.05 compared with I/R + Tyrosol 0.25 mM cells, and e: *p* < 0.05 compared with I/R + Tyrosol 0.50 mM cells.

Two commonly used measures of apoptosis, nuclear condensation (NCI) and caspase-3 activity, were used to evaluate protection of H9c2 cardiomyocytes by tyrosol. The extent of NCI was quantitated using the DNA-binding fluorescent dye DAPI. Figure 2A-a,b shows that control cells and those treated with 0.50 mM tyrosol had nuclei that were relatively large and uniform in terms of both size and morphology. However, I/R cells exhibited obvious nuclear condensation, indicating the occurrence of apoptosis (Figure 2A-c). However, tyrosol at 0.50 mM decreased the extent of NCI in H9c2 cardiomyocytes (Figure 2A-d). Quantitation of the extent of NCI using the Image J software revealed that the proportion of NCI in the I/R group was 33.8% ± 3.2% and tyrosol at 0.10, 0.25, and 0.50 mM attenuated NCI to 20.6% ± 3.3% (*p* < 0.05), 14.8% ± 1.0% (*p* < 0.05), and 12.1% ± 1.4% (*p* < 0.05), respectively (Figure 2B). Significance was displayed between I/R + Tyrosol 0.5 mM and I/R + Tyrosol 0.1 mM. Thus, tyrosol at 0.10, 0.25, and 0.50 mM protected H9c2 cardiomyocytes from I/R-induced NCI.

**Figure 3:**
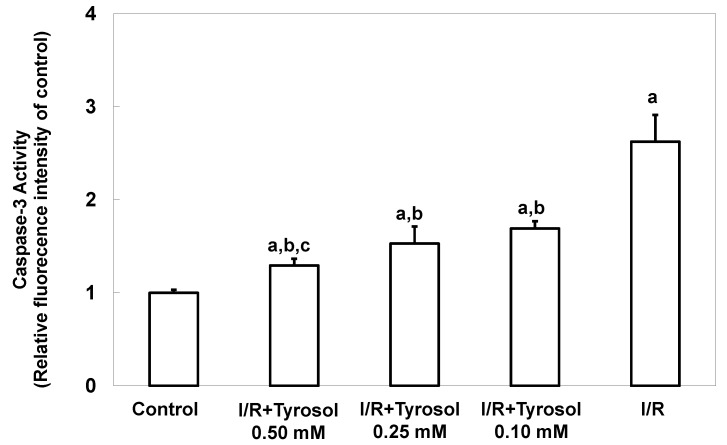
Effect of tyrosol on caspase-3 activity in H9c2 cardiomyocytes during I/R. Cells were pretreated with tyrosol at 0.10, 0.25, or 0.50 mM for 24 h, then subjected to I/R in the presence of the same levels of tyrosol. Caspase-3 activity was assessed using SensoLyte™ (Fremont, CA, USA) Homogeneous Rh110 Caspase-3 Assay Kits. Fluorescence ratios (Ex/Em = 485 nm/538 nm) are shown. Results are expressed as -fold that of control, and means ± SEs (*n* = 3) are shown. a: *p* < 0.05 compared with control, b: *p* < 0.05 compared with I/R cells, c: *p* < 0.05 compared with I/R + Tyrosol 0.10 mM cells.

Caspase-3 activity, another key indicator of apoptosis, was next measured. The caspase-3 activity level was significantly increased by I/R, confirming the occurrence of apoptosis, and tyrosol at 0.10, 0.25, and 0.50 mM significantly inhibited this activity (significance was showed between two treatment groups (I/R + Tyrosol 0.5 mM *vs.* I/R + Tyrosol 0.1 mM), indicating that tyrosol at 0.10, 0.25, and 0.50 mM protected H9c2 cells from I/R-caused caspase-3 activation (Figure 3).

### 2.2. Tyrosol Directly Eliminated I/R-Caused ROS Accumulation in H9c2 Cardiomyocytes

**Figure 4:**
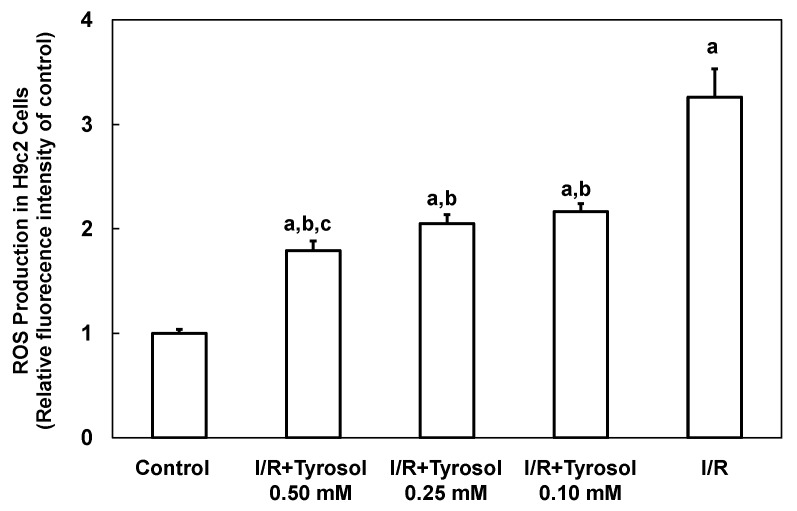
Effect of tyrosol on intracellular ROS production in H9c2 cardiomyocytes during I/R. Cells were incubated with tyrosol (0.10, 0.25, and 0.50 mM) before and during I/R. Cells were also incubated with a DCHF-DA fluorogenic probe (0.1 mM) for 30 min immediately before exposure to I/R and after 24 h of pretreatment with tyrosol. Results are expressed as -fold that of control group and means ± SEs are shown (*n* = 3). a: *p* < 0.05 compared with control, b: *p* < 0.05 compared with I/R cells, c: *p* < 0.05 compared with I/R + Tyrosol 0.10 mM cells.

I/R is implicated in development of a ROS burst and oxidative stress, causing cellular damage. Therefore, intracellular ROS production by H9c2 cardiomyocytes was first examined using the DCF assay. Figure 4 shows that ROS production was greatly increased by I/R (to 3.3 ± 0.3-fold that of the control, *p* < 0.05), indicating that ROS was accumulated in H9c2 cardiomyocytes. Tyrosol (0.10, 0.25, and 0.50 mM) dose-dependently reduced ROS accumulation to 2.2 ± 0.1-(*p* < 0.05), 2.1 ± 0.1-(*p* < 0.05), and 1.8 ± 0.1-(*p* < 0.05) fold that of control, respectively. Significance was shown between I/R + Tyrosol 0.5 mM and I/R + Tyrosol 0.1 mM. Our findings demonstrated that tyrosol at concentrations ranging from 0.10–0.50 mM can protect H9c2 cardiomyocytes against I/R-induced ROS accumulation, thus mitigating damage from oxidative stress.

### 2.3. Tyrosol Exerted Its Protection through Stimulation of Hsp70 Overexpression

**Figure 5:**
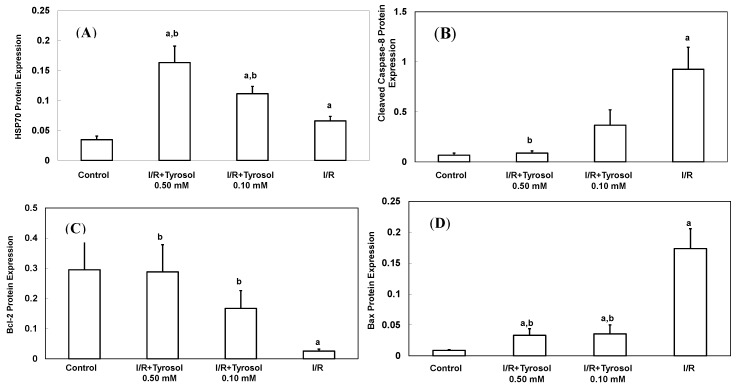

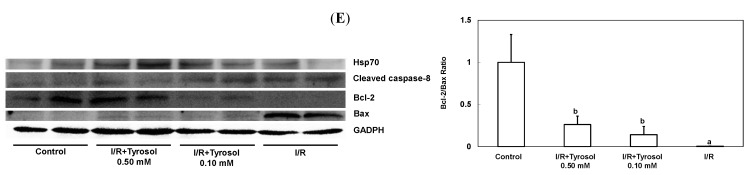
Effect of tyrosol on protein expressions in H9c2 cardiomyocytes during I/R. Cells were incubated with tyrosol (0.10 and 0.50 mM) before and during I/R, and then Western blotting was performed in order to examine the protein expressions in H9c2 cardiomyocytes in the I/R. (**A**) Hsp70; (**B**) cleaved caspase-8; (**C**) Bcl-2; (**D**) Bax; and (**E**) Bcl-2/Bax ratio. Protein bands were visualized by enhanced chemiluminescence reagents and quantified by densitometry with a gel documentation system (Gel-Pro Analyzer, Version 4.0) and shown as density relative to GAPDH. Results were analyzed from six independent experiments (*n* = 6), two lanes in each group represented two replicates in the one membrane, and means ± SEs of the data are shown. a: *p* < 0.05 compared with control, b: *p* < 0.05 compared with I/R cells.

A total of 70 Da of heat shock protein (Hsp70), an inducible protein, was reacted with a variety of stress stimulus including oxidative stress. It protected myocytes from oxidative stress-triggered cell death [39]. To determine the effect of tyrosol on its expression during I/R, Western blotting was adopted (Figure 5A). Our results show that treatment with tyrosol notably enhanced Hsp70 expression in the I/R process, exhibiting the strong stimulating effect of tyrosol on Hsp70 overexpression.

### 2.4. Tyrosol Inhibited Caspase-8 Activation during I/R

Caspase-8 belongs to the family of caspases enzyme, which is responsible for the onset of apoptosis or cell death. In order to examine the effect of tyrosol on caspase-8 activation in I/R, cleaved caspase-8 was investigated by Western blotting (Figure 5B). The results demonstrate that I/R led to caspase-8 activation, while tyrosol displayed significantly inhibitory effect on the activation of caspase-8 in I/R, manifesting protection of tyrosol against I/R-caused cell death via prohibition of caspase-8.

### 2.5. Tyrosol Retained Bcl-2/Bax Ratio in the I/R Injury

The ratio between anti-apoptotic protein (Bcl-2) and proapoptotic protein (Bax), namely Bcl-2/Bax ratio is vital to determination of cell survival or death in the apoptosis response. Therefore, Western blotting assay was conducted to examine Bcl-2 and Bax protein expression in H9c2 cells during I/R injury. Our results show that I/R mitigated Bcl-2 expression and elevated Bax expression, while treatment with tyrosol preserved the Bcl-2 protein level and inhibited the elevation of Bax level (Figure 5C,D), thus collectively resulting in the maintenance of the Bcl-2/Bax ratio in the I/R injury (Figure 5E).

### 2.6. Tyrosol Mediated Protection against Cell Death via JNK and ERK-Dependent Signaling Pathway

**Figure 6:**
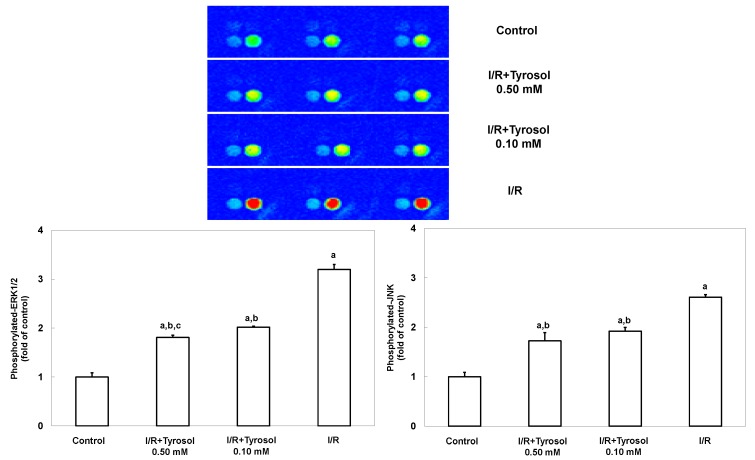
Effect of tyrosol on ERK and JNK activation in H9c2 cardiomyocytes during I/R. Cells were incubated with tyrosol (0.10 and 0.50 mM) before and during I/R. Phosphorylated-ERK and phosphorylated-JNK levels were determined using a MAP kinase phosphoprotein electrochemiluminescent sandwich ELISA assay. Sectoral images exhibiting chemiluminescence are shown, reflecting the levels of p-ERK and p-JNK in the samples. Left and right bright spots represent p-JNK and p-ERK, respectively. Results are expressed as fold that of control group and means ± SEs of the data of three independent experiments are shown. a: *p* < 0.05 compared with control, b: *p* < 0.05 compared with I/R cells, c: *p* < 0.05 compared with I/R + Tyrosol 0.10 mM cells.

JNK and ERK signaling pathways are highly involved with stress stimulus including I/R and oxidative stress, and mediate such stress-caused cell mortality. To investigate the effects of tyrosol on JNK and ERK signaling, MAP kinase phospho-protein electrochemiluminescent sandwich ELISA arrays (Meso Scale Discovery, Gaithersburg, MD) was performed to examine phosphorylated protein levels of JNK and ERK1/2 in H9c2 cells during I/R (Figure 6). We found that I/R stimulated the expression of phosphorylated JNK and ERK1/2, indicating the activation of such two enzymes. However, treatment with tyrosol significantly blunted this I/R-caused activation, being indicative of the inhibitory effect of tyrosol on JNK and ERK signaling pathway during I/R.

### 2.7. Discussion

Tyrosol is a common bioactive compound with beneficial cardiovascular properties. Recent reports have underlined that tyrosol as a natural dietary antioxidant can be ingested in bulk from olive oil and other foods and herbs, signifying universality of tyrosol intake [27,28]. Moreover, tyrosol is accumulated in various organs including heart (highest levels in the heart followed by spleen, kidney, liver and lungs) [36], implying that tyrosol plays considerable roles in these organs. However, it remains unknown whether pharmacological concentration of tyrosol (such as 0.50mM) could be reached in the organs of heart. Therefore, it should be noted that the concentration range (0.10–0.50 mM) used in this study could represent a pharmacological approach rather than that of dietary or nutritional approaches. Nevertheless, purified tyrosol as health supplements may still have wide application in the future.

In the present study, we concentrated on the cytoprotective effect of tyrosol on H9c2 cardiomyocytes in the process of I/R and its underlying molecular mechanisms. Our results identify tyrosol as a promising cytoprotective natural compound as it salvaged cardiomyocyte loss, and protected against the occurrence of apoptosis in the H9c2 cardiomyocytes during I/R process. The myocardial protection of tyrosol is primarily through mitigation of oxidative stress, augmenting expression of Hsp70 and regulation of MAPKs, specifically, inhibiting JNK and ERK activation. Furthermore, it was firmly corroborated with the cytoprotection of tyrosol as it served as a potent agent by preserving Bcl-2, reducing Bax expression, hence rescuing Bcl-2/Bax ratio, inhibiting caspase-8 and caspase-3 activation and nuclear condensation, and leading to the eventual salvation of cardiomyocyte loss. Therefore, we identify a novel mechanism whereby tyrosol could be a potent natural compound against I/R-caused myocardial injury.

Ischemia-reperfusion destruct normal oxidative metabolism, leading to ROS accumulation, thus creating oxidative stress. It is widely recognized that oxidative stress, caused by ROS synthesis, is a principal contributor to the detrimental effect of I/R on the cardiovascular system and a major stimulus that leads to apoptosis or cell death, thus resulting in heart failure and mortality [40]. Inhibition of oxidative stress by antioxidants has been shown to alleviate I/R-induced cardiomyocyte injury and restore heart function. In addition to direct elimination of free radical overproduction, some antioxidants show salvaging effects by interaction with key components and regulators in the lethal pathway.

The present study shows that ROS levels were notably elevated after I/R, indicating ROS accumulation formed in H9c2 cardiomyocytes, while I/R-caused ROS accumulation was significantly inhibited by tyrosol, suggesting a direct ROS eliminating effect by tyrosol. Reports have been shown that tyrosol can improve intracellular antioxidant defense systems in macrophages and exert antioxidant capacity in human endothelial cells [37]. However, evidence for an anti-oxidant activity of tyrosol in H9c2 cardiomyocytes during I/R injury was still lacking.

To our knowledge, we are the first to demonstrate (in the present work) that tyrosol can scavenge ROS synthesized in H9c2 cardiomyocytes during I/R injury, indicating that distribution of tyrosol as an antioxidant in the heart did, to some extent, exert its beneficial effect.

Oxidative stress impaired cardiomyocytes by causing apoptosis and cell death, thus engaging in the development of cardiovascular diseases, and the Bcl-2 family is believed to play a critical role in oxidative stress-triggered apoptosis [41]. The present study shows that treatment with tyrosol retained Bcl-2 protein level and attenuated Bax production in H9c2 cells, exhibiting a net preservation of Bcl-2/Bax ratio. It is well recognized that anti-apoptotic effect of bcl-2 is mainly achieved by counteracting pro-apoptotic protein production of bax digging holes in the outer mitochondria membrane, inhibiting cytochrome c release into cytoplasm, and suppressing cytochrome c-mediated caspase cascade [42,43,44,45]. It should be noted that the ratio between Bcl-2 and Bax, practically, is more critical to determining cell death or survival than either alone [46,47].

Heat shock proteins (Hsp) are one of the notable regulators implicated in the oxidant-caused lethal response, and serve as molecular chaperones to assist in protein folding and actively participate under cell stress to minimize damages imposed on cellular components [48,49]. Substantial reports showed that overproduction of Hsp70 protects embryonic rat heart-derived H9c2 myocytes against simulated ischemia [50], exerts myocardial preservation in the I/R process [51] and augments cardiomyocyte viability under oxidative stress [39]. Likewise, Hsp70 in I/R hearts sustains oxidative metabolism, protects mitochondria from ROS overproduction, stabilizes organelles integrity and functions, thus rescuing the cell from apoptosis or death, and consequently reducing infarct size and myocyte loss [52,53,54,55,56,57]. 

Consistent with the above, our results suggest that one of the mechanisms whereby tyrosol exerts its cytoprotection could be via overexpression of Hsp70, as its enhanced expression was presented in the tyrosol treatment group alongside the amelioration of myocyte loss and apoptosis.

The mitogen-activated protein kinases (MAPKs) are signaling pathways with diverse functions including regulation of cell proliferation, survival, and cell death, and are a ROS downstream gateway to apoptosis. JNK signaling cascade activates inflammation and apoptosis, whereas ERK pathway mediates cellular survival and growth [58,59]. ERK activation rendering cardioprotection has been reported by numerous studies [19,20,21]. However, other evidences indicate that ERK activation also plays a role in myocytes loss. For instance, ERK1/2 activation in hypoxia/reoxygenation leads to the loss of rat cardiomyocytes, while mitigation of elevated ERK cascade restores cell viability [22,23]. Another report showed that the onset of necrosis by I/R in H9c2 cardiomyocytes was preferentially accompanied by ERK activation [24], and ERK activation contributed to doxorubicin-caused apoptosis in cardiomyocytes [25], eventually leading to the loss of myocytes.

The present study shows that tyrosol inhibited activations of both JNK and ERK caused by I/R in H9c2 cardiomyocytes. It could be speculated that the salvaging effect of tyrosol, in addition to its anti-apoptotic effect, was partially attributable to the inhibition of necrosis, therefore, resulting in the strong rescue of myocyte during I/R process.

It is generally accepted that cell fates are largely determined by the interaction of heat shock proteins (Hsp) with key components in the apoptotic pathway. Numerous studies suggested that Hsp70 had an interaction with MAPKs. It has been demonstrated that Hsp70 absence leads to ERK activation in the process of apoptosis [60]. Likewise, inhibition of JNK activation may be involved with the action of Hsp70 [61]. Collectively, those suggest that overproduction of Hsp70 by tyrosol may serve as stress sensor in I/R in the regulation of cell survival via an ERK- and JNK-involved signaling pathway. 

In addition, it has been demonstrated that oxidative stress can trigger the activation of caspase-8, thus leading to the activation of caspase cascade, and finally necrosis or apoptosis [62]. Hsp70 can control apoptosis response through inhibition of multiple steps in caspase cascade including caspase-8 activation [63,64]. The present study shows that tyrosol had an inhibitory effect on the activation of caspase-8 and promoting effect on Hsp70 production, suggesting an interaction of Hsp70 with this key kinase in the apoptosis response by I/R-caused oxidative stress. 

A limitation in this study is that H9c2 cell lines rather than cardiomyocytes were applied in the present study. It should be noted that H9c2 cell line was originated from the embryonic rat heart, quite analogous to cardiomyocytes. Nevertheless, our findings may be further corroborated through cardiomyocytes and cardiac tissues in future works.

**Figure 7:**
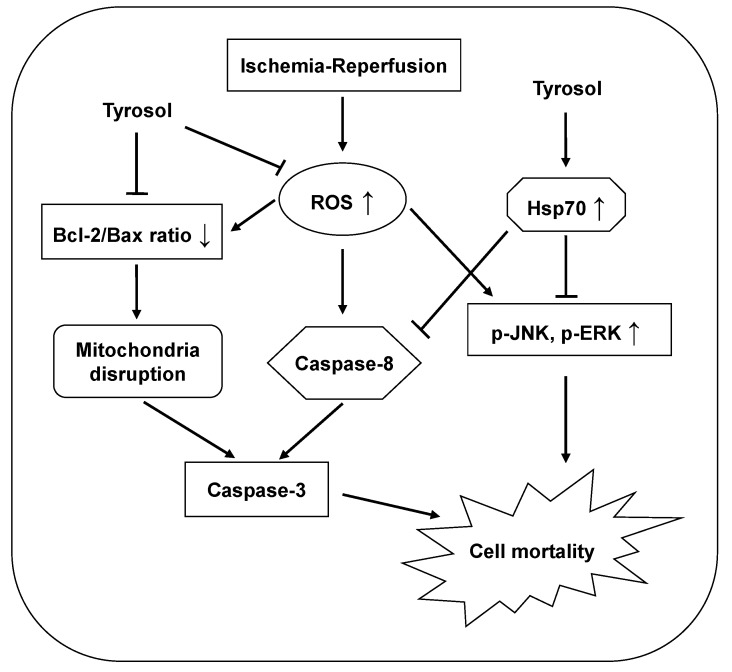
Proposed mechanisms of the cytoprotective effect of tyrosol on I/R-caused mortality in H9c2 cardiomyocytes. Tyrosol protect cardiomyocytes against I/R-caused mortality, at least partially, by inhibition of ROS accumulation, Hsp70-mediated prohibition of JNK and ERK signaling pathway and caspase-8 activation, and restoration of Bcl-2/Bax ratio.

The protective effects of tyrosol are summarized and presented in Figure 7.

## 3. Experimental Section

### 3.1. Cell Culture, Ischemia/Reperfusion Simulation, and Cell Viability Evaluation

H9c2 cell lines were purchased from the American Type Culture Collection and cultured at 37 °C under 5% (v/v) CO_2_ in Dulbecco’s modified Eagle’s medium (DMEM) supplemented with 10% (v/v) heat-inactivated fetal bovine serum and 1% (w/v) of an antibiotic-antimycotic preparation. H9c2 cardiomyocytes were pretreated with tyrosol (Sigma-Aldrich Chemical Co., Oakville, ON, Canada) in DMEM medium for 24 h prior to and during I/R. 

Simulated *ischemia/reperfusion* (I/R) was performed as follows. To induce ischemia, defined as shortage of nutrients and oxygen simultaneously, cells were placed into an “ischemic buffer” and grown under 95% N_2_ and 5% CO_2_ (both v/v) at 37 °C for 1 h in a Modular Incubator Chamber (Billups-Rothenberg, Del Mar, CA, USA). The ischemic buffer (pH 6.3), imitating the situation of lacking nutrients by blood flow obstruction, contained 137 mM NaCl, 15.8 mM KCl, 0.49 mM MgCl_2_, 0.9 mM CaCl_2_, 4 mM HEPES, 10 mM 2-deoxyglucose, 20 mM sodium lactate, and 1 mM sodium dithionite. For subsequent reperfusion, this medium was changed back to DMEM for a 30-min period. Control cells were cultured in DMEM at 37 °C under 5% (v/v) CO_2_ for 90 min. 

Cell viability was evaluated using the 3-(4,5-dimethylthiazol-2-yl)-2,5-diphenyltetrazolium bromide (MTT) assay. Briefly, cells seeded into 96-well plates at 1.0 × 10^4^/well were incubated for approximately 12 h prior to treatment with concentrations of tyrosol for 24 h, then I/R model was conducted in the presence of the same treatment with tyrosol. Subsequently, 20 μL of MTT solution (5 mg/mL) was added to each well to attain a final MTT concentration of 0.5 mg/mL. After incubation for 2 h, the medium was removed and 150-μL DMSO was added to each well. The plate was incubated for a further 10 min and the absorbance levels at 490 nm determined spectrophotometrically. Results are calculated from 10 independent experiments (*n* = 10) for I/R model.

### 3.2. Detection of Apoptosis and Measurements of Caspase-3 and Intracellular ROS

Nuclei were stained with DAPI to evaluate morphological changes in apoptotic cells. Briefly, during reperfusion, H9c2 cardiomyocytes were stained with DAPI for 15 min at 37 °C under 5% (v/v) CO_2_. After washing with cold PBS and fixing in 1.5% (v/v) formalin, cells were examined using an Olympus IX81 microscope (200×) equipped with X-CITE fluorescence illumination (Series 120Q; blue fluorescence). The extent of apoptosis was quantified by calculating the ratio of condensed to total nuclei using the Image J software [65]. Results were shown from the data of five independent experiments (*n* = 5) with 20 random microscopic fields from each sample. Condensed nuclei in the microscopic field were counted by using the Image J software.

Caspase-3 activity was measured as follows. Briefly, cells seeded into 96-well plates at 1.0 × 10^4^/well were cultured overnight prior to incubation with various concentrations of tyrosol. Subsequently, cells were subjected to I/R, followed by addition of 50 μL of reaction buffer and 0.5 μL caspase-3 substrate (Z-DEVD-2-Rh110) in accordance with instructions of a fluorometric caspase-3 assay kit (AnaSpec SensoLyte™, Fremont, CA, USA). After a 4-h incubation in the dark, fluorescence intensities were measured using Ex and Em wavelengths of 485 and 538 nm, respectively. Fluorescence reflected cleavage of the substrate by caspase-3. Results were shown from three independent experiments (*n* = 3).

Intracellular ROS levels were measured using an intracellular ROS assay kit (Cell Biolabs, Inc., San Diego, CA, USA). After seeding at 1.0 × 10^4^/well, cells were cultured in 96-well plates for 12 h prior to addition of tyrosol. Briefly, DCHF-DA stock solution (20 mM) was diluted with medium to a final concentration of 0.1 mM and cells were incubated in this medium for 30 min. After three washes with PBS, cells were subjected to I/R in the dark, and fluorescence intensities measured immediately thereafter using Ex and Em wavelengths of 480 and 530 nm. Results were shown by three independent experiments (*n* = 3).

### 3.3. Western Immunoblotting Analysis

Hsp70, cleaved caspase-8, Bcl-2 and Bax protein levels in the H9c2 cardiomyocytes were determined by Western immunoblotting analysis. Briefly, cell lysates were prepared in a homogenisation buffer [10 mM Tris (pH 7.4), 4 mM EDTA, 1 mM EGTA, 5 mM β-mercaptoethanol, 50 mM NaF, 1 mM Na3VO4, 10 mM Sodium pyrophosphate; containing inhibitors of phosphatases and proteases]. Cell lysates in a SDS sample loading buffer [50 mM Tris-HCL (pH 6.8), 2% SDS, 5% Glycerol, 0.14 M β-mercaptoethanol and 0.004% Bromphenol blue] were denatured in the boiling water for 3 min. The Bradford protein assay was used to determine cellular protein content. Equal amount of cellular proteins (57.6 μg, or 60 μg for Bax) were separated by electrophoresis on a 15% SDS (15% w/v)-PAGE (or 7.5% SDS-PAGE for Hsp70) and then transferred to a nitrocellulose membrane. The membrane was probed with rabbit anti-cleaved caspase-8 polyclonal antibody, rabbit anti-Bcl-2 polyclonal antibody and rabbit anti-Bax polyclonal antibody (Abcam, Cambridge, MA, USA) or a rabbit anti-Hsp70 polyclonal antibody (BioVision, Milpitas, CA, USA). A goat anti-rabbit antibody was used as secondary antibody. Protein bands were visualized by using enhanced chemiluminescence reagents and quantified by densitometry with a gel documentation system (Gel-Pro Analyzer, Version 4.0) and shown as density relative to GAPDH. Results were analyzed from six independent experiments (*n* = 6).

### 3.4. Examinations of JNK and ERK Activation

The extent of JNK and ERK activation was examined by measuring p-JNK and p-ERK1/2 levels using the MAP kinase phosphoprotein electrochemiluminescent sandwich ELISA assay (Meso Scale Discovery, Gaithersburg, MD). Briefly, cell lysates were prepared in sonication buffer [20 mM Tris (pH 7.5), 150 mM NaCl, 1 mM EDTA, 1 mM EGTA, and 1% (v/v) Triton-X100; containing inhibitors of phosphatases and proteases]. The Bradford protein assay was conducted to examine cellular protein content. P-JNK and p-ERK1/2 protein levels were assayed by adding 25-μL volumes of lysate to microplate wells and, 3 h later, detecting antibody and substrate were also added. The intensity of chemiluminescence reflected the p-JNK and p-ERK1/2 levels. Results were analyzed from three independent experiments (*n* = 3).

### 3.5. Statistical Analysis

SPSS for Windows (Version 17.0, SPSS Inc., Chicago, IL, USA) were used. All the data were analyzed by using One-way ANOVA after calculation of normality and homogeneity of variance. If data displayed normal and homogeneous, they were subjected to further examine. If data exhibited nonnormal distribution or unhomogeneous variance, they were lg10 prior to analysis in order to ensure homogeneity of variance. In case transformed data by lg10 is still unhomogeneous, Kruskal-Wallis non-parametric test were used to assess the data between two groups. Statistical tests were performed at *p* = 0.05, and a *p* value <0.05 indicated statistical significance.

## 4. Conclusions

In conclusion, we show that tyrosol exerted a protective effect against the I/R-caused cardiomyocyte apoptosis and mortality by suppressing ROS accumulation, promoting Hsp70 overproduction, blunting JNK and ERK-mediated cascades, elevating Bcl-2/Bax ratio, and inhibiting caspase-8 activation.
